# Motor Competence in Early Childhood Is Positively Associated With Bone Strength in Late Adolescence

**DOI:** 10.1002/jbmr.2775

**Published:** 2016-02-06

**Authors:** Alex Ireland, Adrian Sayers, Kevin C Deere, Alan Emond, Jon H Tobias

**Affiliations:** ^1^ School of Healthcare ScienceManchester Metropolitan UniversityManchesterUK; ^2^ School of Clinical SciencesUniversity of BristolBristolUK; ^3^ School of Social and Community MedicineUniversity of BristolBristolUK

**Keywords:** ALSPAC, MOTOR CONTROL, BODY COMPOSITION, BONE MINERAL DENSITY

## Abstract

The onset of walking in early childhood results in exposure of the lower limb to substantial forces from weight bearing activity that ultimately contribute to adult bone strength. Relationships between gross motor score (GMS), at 18 months and bone outcomes measured at age 17 years were examined in 2327 participants in the Avon Longitudinal Study of Parents and Children (ALSPAC). Higher GMS indicated greater motor competence in weight‐bearing activities. Total hip bone mineral density (BMD) and hip cross‐sectional moment of inertia (CSMI) were assessed from dual‐energy X‐ray absorptiometry (DXA). Bone measures including cortical bone mineral content (BMC), periosteal circumference (PC), cortical thickness (CT), cortical bone area (CBA), cortical BMD (BMD_C_) and cross‐sectional moment of inertia (CSMI) were assessed by peripheral quantitative computed tomography (pQCT) at 50% distal‐proximal length. Before adjustment, GMS was associated with hip BMD, CSMI, and tibia BMC, PC, CT, CBA and CSMI (all *p* < 0.001) but not BMD_C_ (*p* > 0.25). Strongest associations (standardized regression coefficients with 95% CI) were between GMS and hip BMD (0.086; 95% CI, 0.067 to 0.105) and tibia BMC (0.105; 95% CI, 0.089 to 0.121). With the exception of hip BMD, larger regression coefficients were observed in males (gender interactions all *p* < 0.05). Adjustment for lean mass resulted in substantial attenuation of regression coefficients, suggesting associations between impaired motor competence and subsequent bone development are partly mediated by alterations in body composition. In conclusion, impaired motor competence in childhood is associated with lower adolescent bone strength, and may represent a risk factor for subsequent osteoporosis. © 2015 The Authors. *Journal of Bone and Mineral Research* published by Wiley Periodicals, Inc. on behalf of American Society for Bone and Mineral Research (ASBMR).

## Introduction

Bone is strongly influenced by the habitual strains to which it is exposed. Due to the short levers muscles work with, muscular forces are the greatest stressors of bone.^1^ Locomotion results in the largest muscle forces regularly experienced by the body, estimated at three times body weight even during walking.^2^ The attainment of independent walking at around 12 months of age represents the first postnatal exposure of the lower limb bones to these large forces. Accordingly, an increase in bone strength has been observed at ∼15 months of age (ie, shortly after typical walking onset age) that could not be explained by changes in body size.^3^ Recent work has shown a strong relationship between time since onset of walking and bone strength at 15 months, independent of body size or perinatal factors such as birth weight or gestational age.^4^ Children who had been walking for ∼5 months had over 50% greater bone mass and 100% greater torsional bone stiffness at 15 months than children who had yet to walk, despite no association between walking onset age and bone strength at birth. It is plausible, therefore, that early‐life mobility and resultant bone loading may contribute directly to bone strength in childhood and adolescence, thereby representing a risk factor for subsequent osteoporosis.

Motor competence is the ability to carry out motor tasks, and is commonly measured using standardized questionnaires^5^ or clinic‐based assessments.^6^, ^7^ Low bone strength has been observed in adolescents with motor difficulties,^8^ and children, adolescents, and adults with low motor competence.^9^ However, few studies have prospectively collected data on early‐life motor competence and how it influences bone strength in later life. In particular, effects of competence in locomotor skills such as walking and jumping are likely to stress the lower limb bones. Growth velocity in early childhood is greater than at any other time of life,^3^ including the pubertal growth spurt. This period may therefore represent an important time for bone mass accrual. Motor competence may also influence bone strength via changes in physical activity (PA) and/or body composition. Poor motor competence in childhood is associated with reduced PA levels in adolescence,^10^ which is known to affect bone strength.^11^, ^12^ Low motor competence is also associated with greater trunk adiposity.^9^ However, although muscle and bone size are closely related to, even independent of, allometric scaling,^13^ associations between motor competence and muscle size/lean mass (LM) remain unexplored.

In the present study, we examined relationships between early‐life motor competence, and skeletal development in late adolescence, in a large population‐based birth cohort. We hypothesized that greater motor competence in early childhood would be positively associated with bone strength as assessed in later life. In further analyses we aimed to examine to what extent observed relationships could be explained by altered body composition, levels of PA, or motor competence in later childhood.

## Subjects and Methods

### Cohort description

The Avon Longitudinal Study of Parents and Children (ALSPAC) is a geographically‐based birth cohort study investigating influences on health and development of children and young people. Pregnant women resident in the former Avon Health Authority in South West England, having an estimated date of delivery between April 1, 1991, and December 31, 1992, were invited to take part, resulting in a cohort of 14,541 pregnancies and 13,988 children alive at 12 months.^14^ Ethical approval was obtained from the ALSPAC Law and Ethics committee and relevant local ethics committees. Data in ALSPAC are collected via several methods: self‐completion postal questionnaires sent to parents, linkage to computerized records, abstraction from medical records, and from examination of the children at research clinics. The study website (http://www.bristol.ac.uk/alspac/researchers/access) contains details of all the data that are available through a fully searchable data dictionary.

### Exposure variable: Motor competence

An estimate of motor competence in early childhood was obtained at around 18 months of age using a scale developed by ALSPAC including elements derived from the Denver Developmental Screening Test.^5^ Mothers were asked to complete a series of questions as to whether their child regularly, occasionally, or had never completed movements such as walking, climbing, and jumping. These answers were used to calculate a continuous Gross Motor Score (GMS). Age at time of questionnaire completion was also recorded.

### Outcome measures: DXA and peripheral quantitative computed tomography

Participants attending the 17 year research clinic underwent a total body DXA scan using a GE Lunar Prodigy (Madison, WI, USA) in standard scanning mode. Data were analyzed using in‐built GE Lunar enCore software (version 12), from which total body LM and fat mass (FM) were obtained. Total hip bone mineral density (BMD) was obtained from a hip scan. In addition, hip cross‐sectional moment of inertia (CSMI) at the site of minimum femoral neck width was calculated using the manufacturer's automated advanced hip analysis (AHA) software. DXA measures of BMD are influenced by body size during growth. To ensure that observed effects were not simply an artifact of growth, total hip BMC adjusted for total hip bone area was also examined.^15^ Similar results were obtained for hip BMD and size‐adjusted BMC; therefore, only BMD values are reported. A peripheral quantitative computed tomography (pQCT) scan at the 50% tibial site was also taken using an XCT 2000 scanner (Stratec, Pforzheim, Germany). Measurements were analyzed and results exported using the Automated Analysis Tools in Version 6.00B of the software supplied with the machine. Analysis of tibial cortical bone was completed using a threshold of 650 mg/mm^3^, which accurately assesses bone geometry.^16^ Cortical BMC, cortical bone area, and cortical BMD were measured. In addition, periosteal and endocortical circumferences, cortical thickness, and CSMI derived from a circular ring model were recorded.^17^ Muscle area was also measured from pQCT images as described.^18^ Briefly, images were filtered using the in‐built F03F05F05 filter, before a threshold of 30 mg/mm was used to remove fat from the image and calculated total bone area was subtracted. Precision of bone outcomes was assessed from repeated scans using the coefficient of variation (CV). Due to differing rates of compliance the number of individuals with repeated measurements varies between measurement modalities. CV for total hip BMD was 1.2% and hip CSMI 7.5% based on 153 repeat scans. For pQCT outcomes, CVs based on 126 repeat scans were as follows: cortical BMD = 1.1%, cortical BMC = 2.6%, periosteal circumference = 1.5%, endocortical circumference = 3.4%, cortical thickness = 2.1%, and CSMI = 5.8%.

### Other measures

Perinatal variables (birth weight and gestation, length at delivery) were obtained from hospital records, and maternal social class was recorded from a questionnaire completed at 32 weeks gestation. Between 7 and 8 years, children's motor skills were assessed using the ALSPAC Coordination Test (ACT) during an ALSPAC clinic visit, which were used to provide a measure of motor competence in later childhood. The ACT consists of a subset of tests from the Movement Assessment Battery for Children (MABC).^6^ These tests examined balance (heel‐to‐toe walking), ball skills (throwing bean bag into box), and manual dexterity (placing pegs), representing the three domains of coordination identified by principal component analysis of MABC standardization data.^7^ Age at time of testing was recorded. At the age 17 years research clinic, as well as bone measures described in the previous section, mass and height were recorded from which BMI was calculated, and participants who agreed participated in an accelerometer substudy.^19^ Puberty was assessed by self‐completion questionnaires using diagrams based on Tanner staging of pubic hair distribution for both genders. A uniaxial Newtest accelerometer was worn for 7 consecutive days, only removing it when it might get wet or when playing contact sports. They were also asked to record a diary when the monitor was worn. A valid recording was defined as a minimum of 8 hours of recording on 2 separate days, and length of measurement period was recorded. A previous calibration study identified a threshold of 3*g* as representing the transition from moderate impact activities such as brisk walking to high impact activities such as jogging or jumping.^19^ Therefore, the number of counts over 3*g* was calculated using custom‐designed code in Stata 11.2 (StataCorp, College Station, TX, USA). Counts were initially stratified into 33 predefined *g*‐bands, and then separated by the designated threshold. The number of counts was then adjusted for the length of the measurement period for each individual.

### Statistical analyses

Statistical analyses were completed using the R statistical environment (version 3.1.2; https://www.r-project.org/). Children between the 5th and 15th centile of standardized coordination tests are considered to be “at risk” of impairment, whereas a result below the 5th centile is defined as definite motor impairment.^10^ To allow consideration of movement score as a categorical variable, 5th and 15th centile scores for GMS were established for the whole cohort, and applied to partition the subgroups for whom complete cases (including all perinatal, early childhood, and adolescent measures) were obtained. Multiple linear regression was used to examine GMS group differences in bone measures, while adjusting for potential confounders (gender, age at exposure and outcome, and maternal social class). In addition to this analysis, relationships between GMS as a continuous variable and bone outcomes were examined. GMS results were not normally distributed, and this could not be rectified by log or root transformation. However, model residuals were homoskedastic showing normality of error terms. In Model 1, relationships between movement scores and bone outcomes were minimally adjusted for height or tibia length for DXA and pQCT outcomes, respectively, due to the strong association between body size and bone strength. In Model 2, data were further adjusted for maternal social class, gender, and age at exposure and outcome. For Model 3, early‐life factors (gestational age and birth weight) were added to Model 2 as possible further confounders. Models 4 and 5 were also adjusted for adolescent LM and then FM, respectively, which could act as possible mediators. Finally, in Model 6 associations between GMS and bone outcomes were further adjusted for ACT score to assess whether they were mediated by persisting advantages in motor competence in later childhood. In Models 2 through 6, gender*movement score interactions were also examined. Relationships are reported as standardized (beta) regression coefficients indicating standard deviation (SD) increase in bone outcome per SD increase in movement score, and 95% confidence intervals (CIs). Missing data was assumed to be missing at random, and only complete cases were analyzed. Modeling assumptions were checked by inspecting residual plots.

## Results

### Participant characteristics

**Figure 1:**
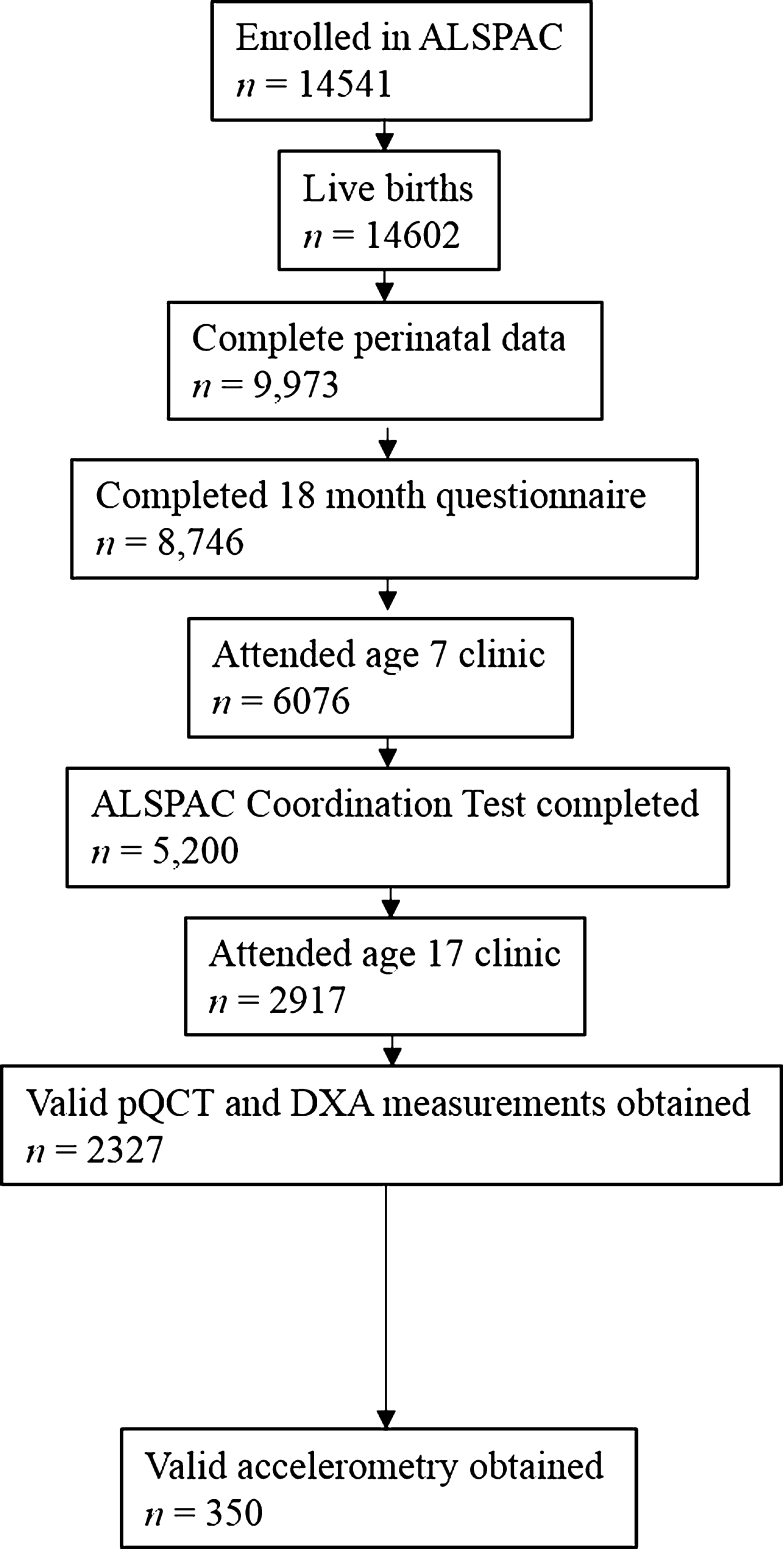
Flow diagram showing participant *n* at each stage of data preparation.

The present analysis is based on 2327 ALSPAC offspring with complete measures, including perinatal data, a GMS score at 18 months, ACT results at age 7 years, and pQCT and DXA measures at age 17.8 years (Fig. 1). Participant characteristics (birth weight, gestation length, and maternal social class) in the present analysis were similar to those for whom complete perinatal data was obtained (*n* = 9973), although there was a greater proportion of males (54%) in the larger cohort than in the present analysis (44%). Males classified as at risk of having impaired motor skills according to GMS had lower body mass at 17 years than healthy children, whereas other characteristics, including birth weight, gestational age, and height at 17 years, were equivalent (Table 1). Females classified as having impaired motor skills had lower gestational age and BMI at 17 years than healthy children.

**Table 1:** Cohort Characteristics in 2327 Participants (1033 Boys) for Groups Separated by Gender and Gross Motor Score Classification at 18 Months

Variable	Healthy	At risk	Impaired
*n*			
Male	862	133	38
Female	1039	196	76
Birth weight (g), mean ± SD			
Male	3476 ± 564	3364 ± 553	3505 ± 491
Female	3376 ± 474	3355 ± 452	3341 ± 509
Gestational age (weeks), mean ± SD			
Male	39.4 ± 1.8	39.1 ± 2.1	39.5 ± 1.7
Female	39.6 ± 1.6	39.5 ± 1.7	39 ± 2.1
Height at 17 years old (cm), mean ± SD			
Male	178.8 ± 6.4	177.7 ± 6	178.7 ± 7
Female	165.4 ± 6.1	165.9 ± 6.6	165.8 ± 7.3
Body mass at 17 years old (kg), mean ± SD			
Male	69.9 ± 10.6	67.2 ± 8.7	66.3 ± 11.2
Female	60.5 ± 9.4	60.3 ± 9.2	58.1 ± 9.1
BMI at 17 years old (kg/m^2^), mean ± SD			
Male	21.9 ± 3	21.3 ± 2.7	20.7 ± 3.1
Female	22.1 ± 3.2	21.9 ± 3.1	21.1 ± 2.8
Gross motor score at 18 months old, mean ± SD			
Male	20.2 ± 1.5	16.3 ± 0.7	11.4 ± 6.4
Female	20.3 ± 1.5	16.2 ± 0.7	11.8 ± 3.2
Maternal social class, n (%)			
I (Professional)			
Male	74 (8.6)	19 (14.3)	5 (16.7)
Female	83 (8.0)	21 (10.7)	6 (9.0)
II (Managerial and technical)			
Male	325 (37.7)	44 (33.1)	17 (56.7)
Female	379 (36.5)	75 (38.3)	25 (37.3)
III (Skilled manual and non‐manual)			
Male	409 (47.4)	62 (46.6)	7 (23.3)
Female	486 (46.8)	90 (45.9)	34 (50.7)
IV (Partly skilled)			
Male	48 (5.6)	7 (5.3)	1 (3.3)
Female	77 (7.4)	10 (5.1)	2 (3.0)
V (Unskilled)			
Male	6 (0.7)	1 (0.8)	0 (0.0)
Female	14 (1.3)	0 (0.0)	0 (0.0)

“At risk” denotes score in lowest 5th to 15th percentile and “Impaired” denotes score in lowest 5th percentile.

### DXA and pQCT outcomes at 17 years old by GMS category

**Figure 2:**
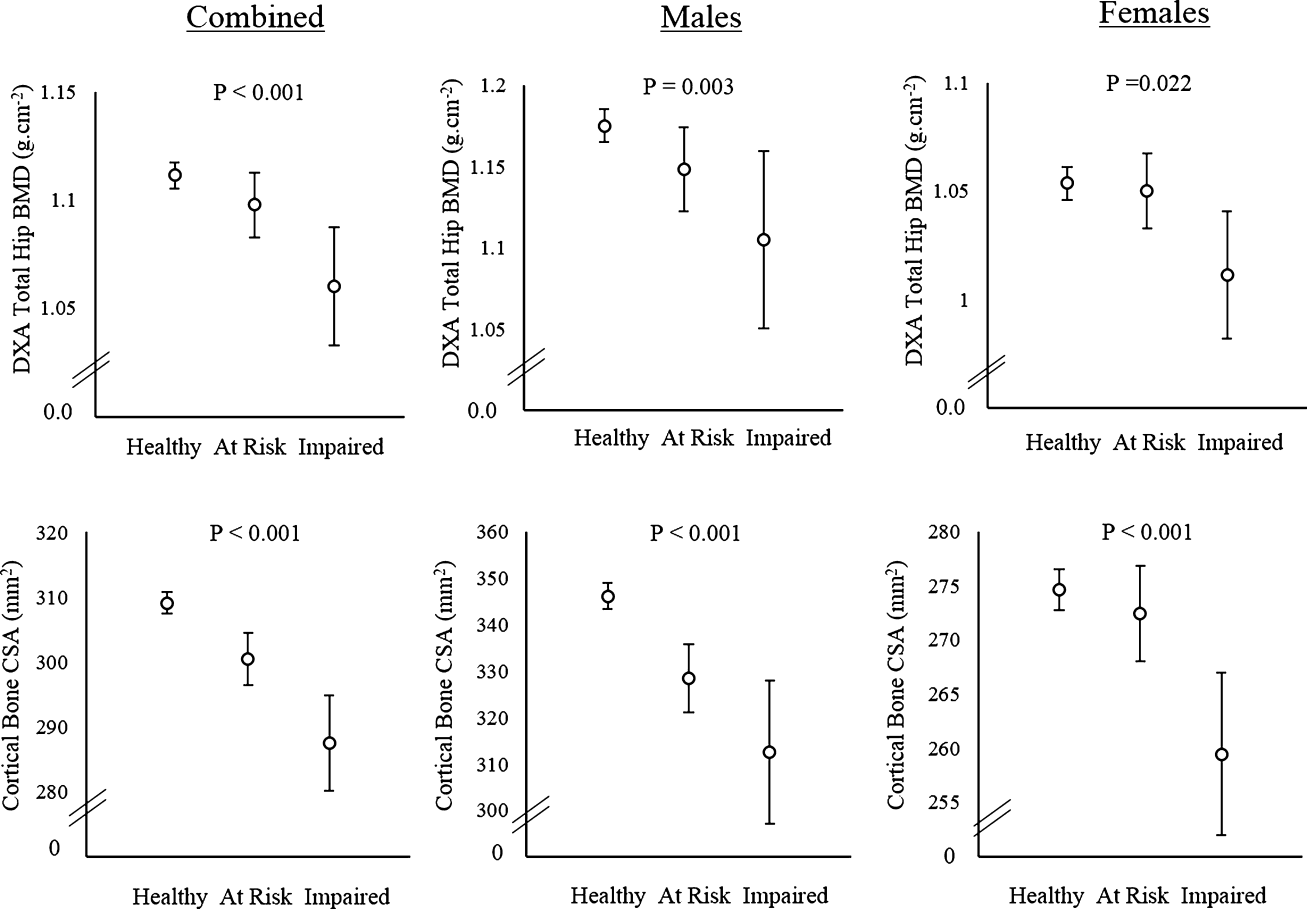
Total hip BMD (upper panels) and cortical bone CSA (lower panels) according to gross motor score at 18 months in males, females, and combined. Results, shown as mean and 95% CI, are adjusted for age at exposure and outcome, height and maternal social class; for combined results data were also adjusted for gender. Values of *p* indicate test for trend for main effect of GMS group. A significant GMS by gender interaction was observed for cortical bone CSA (*p* < 0.001) but not hip BMD (*p* = 0.173).

We compared bone measures at 17 years between healthy, at risk, and impaired groups based on the GMS at 18 months stratified by gender. In unadjusted analyses, compared to controls, males with impaired GMS had a lower hip BMD as measured by DXA, whereas no difference was observed for the at risk group (Table 2). In contrast, male impaired and at risk groups were both lower than male controls for hip CSMI as measured by DXA, and cortical BMC, cortical area, periosteal circumference, cortical thickness, and cortical CSMI as measured by pQCT. Total body LM and muscle CSA were also lower in at risk and impaired groups compared to controls, whereas no differences were seen for total body FM (Table 2). In females, the impaired group had lower hip BMD than controls. Impaired females had lower cortical BMC, cortical CSA, periosteal circumference, cortical thickness and cortical CSMI than both healthy and at risk groups. Muscle CSA was also lower in the impaired group compared to the at risk group and controls, whereas no differences were seen for total body FM or LM. In analyses adjusted for age, gender, height, and maternal social class, negative trends were observed in hip BMD, and in pQCT parameters including periosteal circumference and cortical thickness, on moving from healthy, to at risk, to impaired individuals. Figure 2 shows results of these analyses for hip BMD and cortical BMD, stratified by gender and with data from both genders combined. There was evidence of a movement score by gender interaction (ie, *p* < 0.05) for hip CSMI, and tibia cortical BMC, cortical CSA, periosteal circumference, cortical thickness, and CSMI with greater effects of movement score in males in all cases.

**Table 2:** Unadjusted Bone/Muscle Characteristics Separated by Gender and Gross Motor Score Classification at 18 Months in 2327 Participants (1033 Boys)

Bone/muscle outcome	Healthy	At risk	Impaired
DXA			
Total hip BMD (g/cm^2^)			
Male	1.18 ± 0.16	1.15 ± 0.14	1.11 ± 0.17
Female	1.05 ± 0.12	1.05 ± 0.12	1.01 ± 0.11
Hip CSMI (mm^4^)			
Male	15327 ± 4323	14210 ± 3476	13315 ± 3739
Female	8654 ± 2495	8729 ± 2420	8079 ± 2251
Total body lean mass (kg)			
Male	55 ± 6.1	53.3 ± 5.2	52 ± 6.2
Female	37.7 ± 3.9	37.8 ± 4.1	37 ± 3.9
Total body fat mass (kg)			
Male	11.9 ± 7.3	10.8 ± 6.7	11.3 ± 7.9
Female	19.9 ± 7.4	19.5 ± 6.7	18.3 ± 6.8
pQCT			
Cortical BMC (mg/mm)			
Male	347 ± 46	327 ± 43	312 ± 59
Female	312 ± 39	311 ± 39	294 ± 35
Cortical area (mm^2^)			
Male	384 ± 51	362 ± 48	348 ± 64
Female	274 ± 34	274 ± 34	260 ± 31
Cortical BMD (mg/mm^3^)			
Male	1108 ± 24	1107 ± 25	1114 ± 21
Female	1137 ± 19	1136 ± 19	1131 ± 19
Periosteal circumference (mm)			
Male	77.5 ± 4.8	75.9 ± 5.1	73.8 ± 6.4
Female	68.9 ± 4.2	69 ± 4.3	67.3 ± 4.3
Cortical Thickness (mm)			
Male	5.86 ± 0.67	5.61 ± 0.61	5.5 ± 0.77
Female	5.23 ± 0.54	5.2 ± 0.52	5.07 ± 0.48
Endocortical circumference (mm)			
Male	40.7 ± 5	40.7 ± 5.4	39.3 ± 4.9
Female	36.1 ± 4.4	36.3 ± 4.2	35.5 ± 4.6
CSMI (mm^4^)			
Male	39294 ± 963	36160 ± 9511	33049 ± 10733
Female	24031 ± 5869	24174 ± 6082	21873 ± 5293
Muscle CSA (mm^2^)			
Male	5991 ± 887	5838 ± 781	5654 ± 970
Female	5429 ± 754	5446 ± 792	5227 ± 808

“At risk” denotes score in lowest 5th to 15th percentile; “Impaired” denotes score in lowest 5th percentile.

### DXA and pQCT outcomes at 17 years old by GMS as a continuous variable

To examine the role of further confounders, regression analyses were performed between GMS score as a continuous variable and bone outcomes using a range of models. In Models 1 and 2, equivalent positive relationships were observed between GMS score and hip BMD, hip CSMI, cortical BMC, cortical area, periosteal circumference, cortical thickness, and cortical CSMI to those described above according to GMS classification (Table 3). There was little attenuation after adjustment for gestational age or birth weight, which could also act as possible confounders (Model 3). Subsequently, we explored the role of altered body composition as a possible mediator, by additionally adjusting for LM and FM at age 17 (Models 4 and 5). Associations with hip DXA and pQCT bone outcomes were substantially attenuated by adjustment for LM, as judged by decreases in regression coefficients (Table 4). Nevertheless, with the exception of hip BMD, there was still strong evidence for an association between GMS score and bone parameters. Further adjustment for FM resulted in only minor reductions in coefficients, despite significant relationships between FM and a number of bone outcomes. This was also true when associations were adjusted for FM prior to LM in an alternative Model 4 (results not shown), suggesting that FM did not substantially mediate relationships between GMS and bone outcomes. Model 6 was adjusted for ACT score to study whether GMS and ACT were related to bone outcomes via a common pathway. Although GMS score was associated with ACT score (–0.106; 95% CI, 0.085 to 0.127; *p* < 0.001), adjustment for ACT resulted in only minor attenuation of regression coefficients for associations between GMS and bone outcomes.

**Table 3:** Associations Between Gross Motor Score at 18 Months and DXA‐Derived and pQCT‐Derived Bone Measures in 2327 Participants (1033 Boys)

	Model 1			Model 2				Model 3			
Bone outcome	Beta	95% CI	*p*	Beta	95% CI	*p*	GMS*S	Beta	95% CI	*p*	GMS*S
DXA											
Total hip BMD (g/cm^2^)											
Combined	0.082	(0.045–0.12)	<0.001	0.083	(0.046–0.12)	<0.001	0.163	0.079	(0.042–0.116)	<0.001	0.143
Male	0.100	(–0.02 to 0.221)	0.001	0.107	(0.047–0.168)	<0.001		0.104	(0.043–0.164)	<0.001	
Female	0.075	(0.021–0.129)	0.007	0.077	(0.023–0.132)	0.005		0.072	(0.017–0.126)	0.010	
Hip CSMI (mm^4^)											
Combined	0.071	(0.043–0.1)	<0.001	0.067	(0.04–0.093)	<0.001	<0.001	0.065	(0.039–0.092)	<0.001	<0.001
Male	0.133	(0.079–0.187)	<0.001	0.133	(0.078–0.188)	<0.001		0.128	(0.073–0.182)	<0.001	
Female	0.044	(–0.01 to 0.093)	0.080	0.044	(0–0.094)	0.078		0.048	(–0.002 to 0.098)	0.057	
pQCT											
Cortical BMC (mg/mm)											
Combined	0.113	(0.079–0.146)	<0.001	0.105	(0.075–0.136)	<0.001	0.002	0.102	(0.072–0.132)	<0.001	0.003
Male	0.165	(0.108–0.222)	<0.001	0.168	(0.11–0.226)	<0.001		0.162	(0.105–0.219)	<0.001	
Female	0.101	(0.05–0.151)	<0.001	0.102	(0.052–0.153)	<0.001		0.099	(0.048–0.15)	<0.001	
Cortical area (mm^2^)											
Combined	0.105	(0.073–0.137)	<0.001	0.098	(0.07–0.127)	<0.001	<0.001	0.095	(0.067–0.124)	<0.001	<0.001
Male	0.165	(0.109–0.221)	<0.001	0.168	(0.111–0.224)	<0.001		0.162	(0.106–0.218)	<0.001	
Female	0.091	(0.041–0.14)	<0.001	0.093	(0.043–0.143)	<0.001		0.090	(0.04–0.14)	<0.001	
Cortical BMD (mg/mm^3^)											
Combined	0.009	(–0.03 to 0.044)	0.604	0.015	(–0.02 to 0.046)	0.363	0.008	0.015	(–0.017 to 0.047)	0.359	0.008
Male	–0.037	(–0.09 to 0.021)	0.209	–0.037	(–0.1 to 0.022)	0.217		–0.037	(–0.095 to 0.022)	0.216	
Female	0.072	(0.022 to 0.121)	0.005	0.066	(0.016–0.116)	0.010		0.067	(0.017–0.117)	0.009	
Periosteal circumference (mm)											
Combined	0.097	(0.068–0.126)	<0.001	0.089	(0.063–0.114)	<0.001	0.001	0.085	(0.059–0.11)	<0.001	0.002
Male	0.162	(0.11–0.214)	<0.001	0.165	(0.112–0.218)	<0.001		0.158	(0.106–0.211)	<0.001	
Female	0.080	(0.035–0.126)	<0.001	0.083	(0.037–0.129)	<0.001		0.079	(0.033–0.124)	<0.001	
Cortical thickness (mm)											
Combined	0.098	(0.059–0.136)	<0.001	0.089	(0.053–0.125)	<0.001	0.025	0.088	(0.051–0.124)	<0.001	0.027
Male	0.132	(0.071–0.192)	<0.001	0.133	(0.071–0.194)	<0.001		0.128	(0.067–0.189)	<0.001	
Female	0.071	(0.017–0.125)	0.010	0.073	(0.018–0.127)	0.009		0.071	(0.016–0.126)	0.011	
Endocortical circumference (mm)											
Combined	0.036	(0.002–0.069)	0.038	0.033	(0–0.066)	0.053	0.326	0.029	(0–0.062)	0.083	0.356
Male	0.049	(–0.01 to 0.104)	0.081	0.052	(0–0.108)	0.072		0.049	(–0.007 to 0.105)	0.089	
Female	0.022	(–0.03 to 0.071)	0.369	0.024	(–0.03 to 0.073)	0.343		0.021	(–0.028 to 0.07)	0.402	
CSMI (mm^4^)											
Combined	0.092	(0.062–0.122)	<0.001	0.083	(0.057–0.109)	<0.001	<0.001	0.079	(0.053–0.105)	<0.001	<0.001
Male	0.148	(0.095–0.202)	<0.001	0.151	(0.097–0.204)	<0.001		0.145	(0.092–0.198)	<0.001	
Female	0.073	(0.011–0.134)	0.002	0.074	(0.028–0.12)	0.002		0.071	(0.025–0.117)	0.003	

Data are presented for males, females, and combined gender, and show standardized regression coefficients (Beta), 95% CIs, *p*, and gender interaction (GMS*S). Interaction not examined for Model 1 because data were not adjusted for gender). Adjustments: Model 1: height/tibia length (dependent on whether outcome was derived from DXA or tibial pQCT scans); Model 2: Model 1 + gender, age at exposure, age at outcome, maternal social class; Model 3: Model 1 + gestational age and birth weight.

**Table 4:** Associations Between Gross Motor Score at 18 Months and DXA‐Derived and pQCT‐Derived Bone Measures in 2327 Participants (1033 Boys)

	Model 4				Model 5				Model 6			
Bone outcome	Beta	95% CI	*p*	GMS*S	Beta	95% CI	*p*	GMS*S	Beta	95% CI	*p*	GMS*S
DXA												
Total hip BMD (g/cm^2^)												
Combined	0.033	(0–0.066)	0.053	0.730	0.031	(0–0.064)	0.064	0.752	0.025	(−0.008 to 0.058)	0.131	0.840
Male	0.023	(−0.03–0.075)	0.378		0.021	(−0.03 to 0.071)	0.406		0.017	(−0.034 to 0.069)	0.514	
Female	0.049	(0–0.1)	0.059		0.047	(0–0.097)	0.069		0.042	(−0.009 to 0.093)	0.105	
Hip CSMI (mm^4^)												
Combined	0.032	(0.008–0.056)	0.009	0.016	0.030	(0.007–0.053)	0.013	0.012	0.027	(0.003–0.05)	0.028	0.009
Male	0.058	(0.011–0.106)	0.017		0.057	(0.009–0.104)	0.020		0.053	(0.005–0.1)	0.030	
Female	0.028	(−0.02–0.075)	0.237		0.023	(−0.02 to 0.068)	0.315		0.020	(−0.025 to 0.066)	0.378	
pQCT												
Cortical BMC (mg/mm)												
Combined	0.084	(0.056–0.113)	<0.001	0.005	0.083	(0.055–0.111)	<0.001	0.003	0.076	(0.048–0.104)	<0.001	0.002
Male	0.079	(0.033–0.125)	<0.001		0.075	(0.03–0.12)	0.001		0.070	(0.026–0.115)	0.002	
Female	0.068	(0.023–0.113)	0.003		0.060	(0.017–0.103)	0.007		0.056	(0.013–0.1)	0.012	
Cortical area (mm^2^)												
Combined	0.080	(0.053–0.107)	<0.001	<0.001	0.079	(0.053–0.106)	<0.001	<0.001	0.072	(0.046–0.099)	<0.001	<0.001
Male	0.087	(0.04–0.133)	<0.001		0.083	(0.038–0.128)	<0.001		0.078	(0.033–0.123)	<0.001	
Female	0.061	(0.017–0.106)	0.007		0.053	(0.01–0.096)	0.015		0.050	(0.007–0.093)	0.023	
Cortical BMD (mg/mm^3^)												
Combined	0.015	(−0.02–0.046)	0.374	0.007	0.014	(−0.02 to 0.046)	0.387	0.008	0.016	(−0.016 to 0.048)	0.337	0.008
Male	–0.056	(−0.011–0.003)	0.063		0.057	(0.116–0.001)	0.055		–0.054	(−0.112 to 0.004)	0.071	
Female	0.070	(0.02–0.12)	0.007		0.069	(0.019–0.119)	0.007		0.086	(0.023–0.15)	0.008	
Periosteal circumference (mm)												
Combined	0.068	(0.045–0.091)	<0.001	0.004	0.067	(0.044–0.091)	<0.001	0.003	0.061	(0.038–0.085)	<0.001	0.002
Male	0.087	(0.043–0.131)	<0.001		0.084	(0.04–0.127)	<0.001		0.079	(0.036–0.123)	<0.001	
Female	0.054	(0.013–0.095)	0.010		0.048	(0.009–0.088)	0.017		0.043	(0.003–0.083)	0.034	
Cortical thickness (mm)												
Combined	0.075	(0.039–0.111)	<0.001	0.049	0.074	(0.038–0.11)	<0.001	0.043	0.069	(0.033–0.105)	<0.001	0.035
Male	0.062	(0.007–0.118)	0.029		0.060	(0.004–0.115)	0.035		0.056	(0.001–0.112)	0.049	
Female	0.050	(0–0.102)	0.061		0.045	(−0.01 to 0.097)	0.083		0.045	(−0.007 to 0.097)	0.088	
Endocortical circumference (mm)												
Combined	0.020	(−0.01–0.053)	0.230	0.482	0.020	(−0.01 to 0.053)	0.234	0.474	0.017	(−0.016 to 0.05)	0.315	0.447
Male	0.034	(−0.02–0.09)	0.245		0.032	(−0.02 to 0.089)	0.263		0.031	(−0.026 to 0.088)	0.284	
Female	0.013	(−0.04–0.062)	0.592		0.011	(−0.04 to 0.06)	0.647		0.069	(−0.42 to 0.558)	0.782	
CSMI (mm^4^)												
Combined	0.061	(0.038–0.084)	<0.001	<0.001	0.060	(0.037–0.083)	<0.001	<0.001	0.053	(0.03–0.076)	<0.001	<0.001
Male	0.067	(0.023–0.11)	0.003		0.063	(0.02–0.105)	0.004		0.058	(0.016–0.1)	0.007	
Female	0.043	(0.003–0.083)	0.035		0.036	(0–0.074)	0.062		0.031	(−0.008 to 0.069)	0.117	

Data are presented for males, females, and combined gender show standardized regression coefficients (Beta), 95% CIs, *p*, and gender interaction (GMS*S). Adjustments: Model 4: Model 3 (height/tibia length [dependent on whether outcome was derived from DXA or tibial pQCT scans], gender, age at exposure, age at outcome, maternal social class, gestational age, birth weight) + lean mass at 17 years old; Model 5: Model 4 + fat mass at 17 years old; Model 6: Model 5 + ALSPAC Coordination Test (ACT) Score at 7 years old.

All models showed evidence of a gender interaction for all parameters apart from hip BMD. This consisted of a stronger association in males compared to females (Tables 3 and 4). For example, in Model 6, male coefficient for periosteal circumference (beta coefficient 0.079; 95% CI, 0.036 to 0123) was much larger than the respective female coefficient (0.043; 95% CI, 0.003 to 0.083). An exception was cortical BMD, which showed negative and positive associations in males and females, respectively (in Model 6, males: –0.054 [95% CI, –0.112 to –0.004]; females: 0.086 [95% CI, 0.023 to 0.15]).

When grouped by ACT centile at 7 years, DXA and pQCT variables tended to be greater in healthy than at risk and impaired individuals (data not shown). Regression analyses between ACT score (as a continuous variable) and bone outcomes revealed similar associations to those seen for GMS score, with the exception that beta coefficients were negative reflecting the fact that individuals with impaired coordination had higher scores (Supporting Tables  1 and 2). Only adjustment for LM in Model 4 resulted in substantial attenuation of associations between ACT and bone outcomes, and all models showed evidence of a gender interaction, consisting of a stronger association in males compared to females for all parameters; apart from cortical BMD, which showed null associations throughout.

### Exploration of further models: accelerometry subcohort

Finally, we examined whether PA plays a role in mediating the association between motor and skeletal development, by further analyzing models adjusted for PA levels, based on the subset of 350 participants with matching accelerometry data obtained at the time of the age 17 years bone assessments. Number of impacts >3*g* per minute of accelerometer use, representing higher levels of impact to which the skeleton preferentially responds, were used as our primary PA measure. There was no gender*GMS interaction on PA (*p* = 0.31); therefore, data from both genders were pooled. GMS at 18 months was positively associated with PA at age 17 years (0.145; 95% CI, 0.01 to 0.198; *p* = 0.007) (model 1). PA at 17 years old was also associated with hip BMD at 17 years old in this subset (0.133; 95% CI, 0.083 to 0.184; *p* = 0.008). GMS was associated with hip BMD (0.122; 95% CI, 0.072 to 0.172; *p* = 0.015) as in the main dataset (results shown for Model 2). The latter association was substantially attenuated by further adjustment for body composition (ie, Model 5) as in the main dataset (0.052; 95% CI, 0.006 to 0.097; *p* = 0.259), but only weakly attenuated by adjustment for PA (0.105; 95% CI, 0.055 to 0.155; *p* = 0.037).

## Discussion

Having investigated associations between motor competence in early childhood and bone strength in adolescence, greater motor competence at 18 months was found to be associated with higher hip BMD and predicted bone strength at 17 years, particularly in males. These associations were partially, but not completely, attenuated by adjustment for body composition (particularly LM). In contrast, the association between motor competence and hip BMD was only marginally attenuated by adjusting for PA. Hence, differences in body composition, as opposed to PA, appear to partly mediate the observed relationships between motor skills and bone development.

That early‐life motor competence was positively associated with bone strength in adolescence extends previous work,^4^ in which bone strength advantages in early‐walking children was attributed to greater habitual loading of the lower limbs. Habitual loading is a primary determinant of bone strength, with prolonged disuse^20^, ^21^ and exercise/PA^4^, ^22^ resulting in up to 50% loss and gain of tibial bone mass, respectively. Greater diaphyseal BMC was observed at the mid‐tibia, which appeared to result from greater periosteal circumference (and hence cortical area and moment of inertia), rather than differences in BMD. A similar pattern has been observed in regular exercise,^22^, ^23^, ^24^ high levels of vigorous PA,^11^, ^12^, ^25^ and in early locomotion,^4^ supporting a role of altered loading in effects of motor competence on bone.

PA assessed by accelerometry is an indicator of habitual loading of the bones and is positively associated with bone strength,^11^, ^12^, ^25^, ^26^, ^27^ particularly in males.^27^ The greatest loads experienced by bone result from internal muscle forces^1^; accordingly, strong relationships also exist between muscle size (as an indicator of muscle strength) and bone strength^13^, ^28^ even when adjusted for body size. In boys, childhood motor competence is positively associated with PA in later childhood^10^ and body composition.^9^ This suggests that early‐life motor competence may influence adolescent bone strength through effects on PA and body composition. We explored these relationships further by adjustment of regression models for LM/muscle CSA, FM, and—in a subcohort—PA assessed by accelerometry. GMS was positively associated with LM as measured by DXA, particularly in males, although no association was seen with FM. Adjustment for LM or muscle size resulted in substantial attenuation of relationships between early‐life motor competence and bone strength, whereas adjustment for FM did not substantially influence these associations. Further adjustment for PA resulted in only weak attenuation of beta coefficients, suggesting that PA assessed by accelerometry does not play a primary role in mediating this relationship. One caveat is that PA is strongly related to body composition, which is in turn an important determinant of bone strength, with positive effects of both LM^13^ and FM^29^ observed in children. Therefore, it may be that muscle size is acting as an indicator of physical activity. Given these complex interrelationships between early motor development, LM, PA, and bone outcomes, caution is needed when proposing causal pathways, particularly given the limited number of participants in whom information on all these variables was available. Associations between movement score and bone outcomes could also be mediated by effects on growth and development. However, this seems unlikely because in addition analyses in 1655 participants (700 males) for whom details of pubertal development at 13.5 years were available there was no effect of GMS or GMS by gender interaction.

Effects of early‐life motor competence on bone strength in adolescence were generally more pronounced in males than females. These findings are consistent with a previous report in ALSPAC that effects of early‐life motor competence on PA in later childhood appear limited to males.^10^ Similarly, the relationship between early‐life motor competence and LM in adolescents, which partly mediates the relationship with bone strength, was considerably stronger in males compared to females (results not shown). Taken together, these results suggest that motor competence has a greater influence on habitual loading in males. Resultant effects on bone may be compounded by the fact that the skeleton of adolescent males appears more responsive to high impact activity^27^ and long‐term exercise^22^, ^23^ compared to females. However, skeletal growth and maturation is slower in boys compared to girls,^30^ and cortical BMD at age 17 years is lower in males compared to females consistent with the fact that males are yet to complete cortical consolidation.^27^ Therefore, gender differences in effects of motor competence on bone strength could theoretically represent effects on the rate of maturation, rather than final bone mass achieved. Repeat DXA measures are currently being obtained in this cohort at age 24 years, and examination of whether associations with GMS score persist into adulthood should be possible to address this possibility.

Although relationships between motor competence and bone were observed for both motor skill scores examined, associations were generally stronger for GMS at 18 months than ACT score at 7 years. This is likely to reflect the fact that GMS at 18 months is related to locomotory tasks likely to stress the lower limb bones, whereas ACT score at 7 years also measures upper limb and fine motor tasks. Furthermore, adjustment for ACT score did not substantially attenuate relationships between GMS and bone outcomes, suggesting the two scores represent different aspects in terms of how motor competence influences bone development.

Bone mass accrual rates slow in mid‐late teens and bone mass typically peaks in the third decade^31^, ^32^ and has a sizeable effect on fracture risk in later life.^33^ Therefore, attainment of high bone strength by the end of adolescence is important for the prevention of fractures later in life.^34^ Growth velocity and periosteal apposition in infancy is even greater than that during adolescence.^3^, ^35^ Recent evidence has shown that greater periosteal circumference in early life is maintained for several decades,^36^ but that the ability to increase bone strength via exercise following skeletal maturity is greatly diminished—particularly at epiphyseal sites.^23^ Hence, this early‐life growth spurt could also represent an important period in the acquisition of bone mass, and a period when exercise effects on bone have pronounced long‐term effects. This is supported by studies showing strong effects of early‐life PA on bone, whereby a 1.0 SD difference in age of independent walking was associated with a 0.5 SD difference in bone mass and torsional strength.^4^ Similar large effect sizes were observed between the different motor competence groups in this study. Motor competence below the 5th centile at 18 months was associated with a 0.45 SD lower total hip BMD in males, and 0.25 SD in females compared to the cohort mean. Fracture risk approximately doubles for each 1.0 SD decrease in BMD; therefore, males and females below the 5th centile could be assumed to have a 35% to 40% and ∼20% greater fracture risk, respectively. However, the amount of variation in bone strength explained by variation in GMS in Model 6, as reflected by beta coefficients, was relatively small. This suggests that other factors such as associated differences in LM explains most of the differences in bone strength according to GMS score.

One limitation of our study is missing data for exposures and outcomes, which may have underestimated the strength of the associations shown. The participants included in this study are only a selected subgroup of the whole cohort; therefore, the extent to which these findings can be extended to the wider population may be limited. The ALSPAC cohort is a longitudinal observational study, and hence causality cannot be attributed for observed associations. In addition, there remains the risk of residual confounding, that factors not included in the analysis influence exposures and outcomes. A further limitation is that birth weight and gestational age were obtained from hospital records rather than according to a standard protocol during study visits. In addition, pQCT is subject to partial volume effects, which can affect cortical BMD measurements. However, in further analyses where a corrective algorithm^17^ was applied, results were unchanged. A further limitation is that we were unable to examine how motor competence affects trabecular bone because scans were only performed at the mid‐tibia.

In conclusion, we found that impaired motor competence in early life was associated with reduced bone strength in adolescence. Whereas pediatric conditions resulting in delayed motor development are known to be associated with impaired bone development, our results suggest a similar relationship may exist in the wider population. Further studies are justified to examine whether early‐life motor competence represents an independent risk factor for osteoporosis, by investigating whether an equivalent relationship exists with bone outcomes in later life.

## Disclosures

All authors state that they have no conflicts of interest.

## Supporting information

Supporting Information.
