# Supplementary material for: Motor Competence in Early Childhood Is Positively Associated With Bone Strength in Late Adolescence
Source: J Bone Miner Res. 2016 Feb 6;31(5):1089–98. doi: 10.1002/jbmr.2775 (PMC4864944; doi:10.1002/jbmr.2775)
Supplement: Supplementary file 1 — Supporting Information. [file JBMR-31-1089-s001.doc]

**Supporting** Table 1

| Bone Outcome | | Sex | Model 1 | | | Model 2 | | | | Model 3 | | | |
| --- | --- | --- | --- | --- | --- | --- | --- | --- | --- | --- | --- | --- | --- |
|  |  |  | Beta | 95%CI | P | Beta | 95%CI | P | GMS*S | Beta | 95%CI | P | GMS*S |
| DXA | Total Hip BMD (g/cm2) | Combined | -0.072 | (-0.11--0.035) | <0.001 | -0.077 | (-0.114--0.04) | <0.001 | 0.003 | -0.075 | (-0.112--0.038) | <0.001 | 0.004 |
|  |  | Males | -0.136 | (-0.196--0.076) | <0.001 | -0.133 | (-0.194--0.073) | <0.001 |  | -0.128 | (-0.189--0.067) | <0.001 |  |
|  |  | Females | -0.036 | (-0.09-0.018) | 0.191 | -0.035 | (0.019--0.089) | 0.202 |  | -0.036 | (-0.091-0.019) | 0.199 |  |
|  | Hip CSMI (mm4) | Combined | -0.037 | (-0.066--0.009) | 0.011 | -0.049 | (-0.075--0.022) | <0.001 | <0.001 | -0.047 | (-0.073--0.02) | <0.001 | <0.001 |
|  |  | Males | -0.113 | (-0.168--0.058) | <0.001 | -0.113 | (-0.168--0.058) | <0.001 |  | -0.107 | (-0.162--0.052) | <0.001 |  |
|  |  | Females | -0.012 | (-0.059-0.035) | 0.616 | -0.013 | (-0.061-0.035) | 0.599 |  | -0.013 | (-0.062-0.036) | 0.602 |  |
| pQCT | Cortical BMC (mg.mm-1) | Combined | -0.076 | (-0.109--0.043) | <0.001 | -0.076 | (-0.106--0.046) | <0.001 | <0.001 | -0.072 | (-0.102--0.042) | <0.001 | <0.001 |
|  |  | Males | -0.153 | (-0.21--0.096) | <0.001 | -0.153 | (-0.21--0.096) | <0.001 |  | -0.143 | (-0.2--0.086) | <0.001 |  |
|  |  | Females | -0.043 | (-0.11--0.035) | 0.100 | -0.041 | (-0.11--0.035) | 0.113 |  | -0.039 | (-0.11--0.035) | 0.131 |  |
|  | Cortical Area (mm2) | Combined | -0.076 | (-0.108--0.044) | <0.001 | -0.076 | (-0.105--0.047) | <0.001 | <0.001 | -0.071 | (-0.099--0.043) | <0.001 | <0.001 |
|  |  | Males | -0.157 | (-0.213--0.101) | <0.001 | -0.158 | (-0.214--0.102) | <0.001 |  | -0.149 | (-0.205--0.093) | <0.001 |  |
|  |  | Females | -0.042 | (-0.093-0.009) | 0.104 | -0.040 | (-0.09-0.01) | 0.118 |  | -0.038 | (-0.088-0.012) | 0.135 |  |
|  | Cortical BMD (mg.mm-3) | Combined | 0.016 | (-0.018-0.05) | 0.361 | 0.016 | (-0.016-0.048) | 0.323 | 0.129 | 0.016 | (-0.015-0.047) | 0.313 | 0.127 |
|  |  | Males | 0.049 | (0.043-0.055) | 0.100 | 0.050 | (0.044-0.056) | 0.091 |  | 0.051 | (-0.008-0.11) | 0.089 |  |
|  |  | Females | -0.010 | (-0.015-0.005) | 0.709 | -0.009 | (-0.059-0.041) | 0.726 |  | -0.008 | (-0.055-0.039) | 0.739 |  |
|  | Periosteal Circumference (mm) | Combined | -0.072 | (-0.101--0.042) | <0.001 | -0.071 | (-0.045--0.097) | <0.001 | 0.002 | -0.066 | (-0.092--0.04) | <0.001 | 0.003 |
|  |  | Males | -0.146 | (-0.198--0.094) | <0.001 | -0.147 | (-0.2--0.094) | <0.001 |  | -0.136 | (-0.188--0.084) | <0.001 |  |
|  |  | Females | -0.052 | (-0.098--0.006) | 0.028 | -0.051 | (-0.097--0.005) | 0.029 |  | -0.047 | (-0.092--0.002) | 0.042 |  |
|  | Cortical Thickness (mm) | Combined | -0.059 | (-0.097--0.021) | 0.003 | -0.059 | (-0.095--0.022) | 0.002 | 0.002 | -0.057 | (-0.094--0.02) | 0.002 | 0.003 |
|  |  | Males | -0.122 | (-0.183--0.061) | <0.001 | -0.125 | (-0.186--0.064) | <0.001 |  | -0.118 | (-0.179--0.057) | <0.001 |  |
|  |  | Females | -0.013 | (-0.068-0.042) | 0.644 | -0.011 | (-0.067-0.045) | 0.700 |  | -0.012 | (-0.069-0.044) | 0.677 |  |
|  | Endocortical Circumference (mm) | Combined | -0.037 | (-0.071--0.003) | 0.033 | -0.036 | (-0.069--0.003) | 0.003 | 0.834 | -0.032 | (-0.065-0.001) | 0.060 | 0.936 |
|  |  | Males | -0.042 | (-0.098-0.014) | 0.139 | -0.040 | (-0.096-0.016) | 0.161 |  | -0.036 | (-0.092-0.02) | 0.212 |  |
|  |  | Females | -0.040 | (-0.089-0.009) | 0.111 | -0.041 | (-0.09-0.008) | 0.101 |  | -0.037 | (-0.086-0.012) | 0.140 |  |
|  | CSMI (mm4) | Combined | -0.079 | (-0.109--0.049) | <0.001 | -0.079 | (-0.105--0.053) | <0.001 | <0.001 | -0.074 | (-0.1--0.048) | <0.001 | <0.001 |
|  |  | Males | -0.160 | (-0.213--0.107) | <0.001 | -0.161 | (-0.214--0.108) | <0.001 |  | -0.151 | (-0.204--0.098) | <0.001 |  |
|  |  | Females | -0.056 | (-0.01--0.102) | 0.018 | 0.055 | (0.009-0.101) | 0.020 |  | -0.051 | (-0.097--0.005) | 0.029 |  |

Associations between ALSPAC Coordination Test (ACT) score and DXA and pQCT-derived bone measures in 1033 boys and 1294 girls. Data are presented for males, females and combined and shows standardised regression coefficients (Beta), 95% CIs, *P* and sex interaction and sex interaction (GMS*S). Note: interaction not examined for Model 1 as data were not adjusted for sex. Model 1: height/tibia length (dependent on whether outcome was derived from DXA or tibial pQCT scans); model 2: model 1 + age at exposure, age at outcome, maternal social class; model 3: model 2 + gestational age and birth weight.

**Supporting Table 2**

| Bone Outcome | | Sex | Model 4 | | | | Model 5 | | | | Model 6 | | | |
| --- | --- | --- | --- | --- | --- | --- | --- | --- | --- | --- | --- | --- | --- | --- |
|  |  |  | Beta | 95%CI | P | GMS*S | Beta | 95%CI | P | GMS*S | Beta | 95%CI | P | GMS*S |
| DXA | Total Hip BMD (g/cm2) | Combined | -0.052 | (-0.085--0.019) | 0.002 | 0.129 | -0.058 | (-0.09--0.025) | <0.001 | 0.094 | -0.055 | (-0.088--0.022) | 0.001 | 0.008 |
|  |  | Males | -0.076 | (-0.081--0.071) | 0.004 |  | -0.082 | (-0.133--0.031) | 0.002 |  | -0.081 | (-0.132--0.03) | 0.002 |  |
|  |  | Females | -0.034 | (-0.085-0.017) | 0.188 |  | -0.040 | (-0.09-0.01) | 0.118 |  | -0.034 | (-0.084-0.016) | 0.183 |  |
|  | Hip CSMI (mm4) | Combined | -0.030 | (-0.053--0.007) | 0.012 | 0.004 | -0.037 | (-0.06--0.014) | 0.002 | 0.002 | -0.035 | (-0.058--0.011) | 0.004 | 0.001 |
|  |  | Males | -0.064 | (-0.111--0.017) | 0.008 |  | -0.073 | (-0.12--0.026) | 0.003 |  | -0.070 | (-0.117--0.023) | 0.004 |  |
|  |  | Females | -0.011 | (-0.056-0.034) | 0.633 |  | -0.023 | (-0.069-0.023) | 0.322 |  | -0.020 | (-0.065-0.025) | 0.387 |  |
| pQCT | Cortical BMC (mg.mm-1) | Combined | -0.070 | (-0.098--0.042) | <0.001 | <0.001 | -0.075 | (-0.352-0.202) | <0.001 | <0.001 | -0.068 | (-0.095--0.04) | <0.001 | <0.001 |
|  |  | Males | -0.069 | (-0.115--0.023) | 0.003 |  | -0.081 | (-0.126--0.037) | <0.001 |  | -0.077 | (-0.121--0.033) | <0.001 |  |
|  |  | Females | -0.025 | (-0.11--0.035) | 0.276 |  | -0.036 | (-0.11--0.035) | 0.105 |  | -0.029 | (-0.11--0.035) | 0.196 |  |
|  | Cortical Area (mm2) | Combined | -0.069 | (-0.096--0.042) | <0.001 | <0.001 | -0.073 | (-0.099--0.047) | <0.001 | <0.001 | -0.066 | (-0.329-0.197) | <0.001 | <0.001 |
|  |  | Males | -0.078 | (-0.124--0.032) | <0.001 |  | -0.089 | (-0.134--0.044) | <0.001 |  | -0.085 | (-0.13--0.04) | <0.001 |  |
|  |  | Females | -0.023 | (-0.068-0.022) | 0.314 |  | -0.032 | (-0.075-0.011) | 0.141 |  | -0.026 | (-0.069-0.017) | 0.238 |  |
|  | Cortical BMD (mg.mm-3) | Combined | 0.016 | (-0.015-0.047) | 0.311 | 0.125 | 0.014 | (-0.017-0.045) | 0.376 | 0.138 | 0.016 | (-0.016-0.048) | 0.327 | 0.145 |
|  |  | Males | 0.068 | (0.01-0.126) | 0.022 |  | 0.064 | (0.005-0.123) | 0.032 |  | 0.061 | (0.002-0.12) | 0.042 |  |
|  |  | Females | -0.010 | (-0.06-0.04) | 0.697 |  | -0.011 | (-0.061-0.039) | 0.665 |  | -0.003 | (-0.008-0.002) | 0.922 |  |
|  | Periosteal Circumference (mm) | Combined | -0.063 | (-0.087--0.04) | <0.001 | 0.008 | -0.066 | (-0.089--0.043) | <0.001 | 0.017 | -0.060 | (-0.083--0.037) | <0.001 | 0.003 |
|  |  | Males | -0.072 | (-0.117--0.028) | 0.002 |  | -0.081 | (-0.125--0.037) | <0.001 |  | -0.076 | (-0.119--0.033) | <0.001 |  |
|  |  | Females | -0.035 | (-0.075-0.005) | 0.088 |  | -0.044 | (-0.084--0.004) | 0.030 |  | -0.038 | (-0.077-0.001) | 0.058 |  |
|  | Cortical Thickness (mm) | Combined | -0.054 | (-0.089--0.019) | 0.003 | 0.007 | -0.058 | (-0.093--0.023) | 0.001 | 0.005 | -0.051 | (-0.086--0.016) | 0.005 | 0.003 |
|  |  | Males | -0.058 | (-0.113--0.003) | 0.040 |  | -0.065 | (-0.12--0.01) | 0.022 |  | -0.062 | (-0.118--0.006) | 0.029 |  |
|  |  | Females | -0.001 | (-0.036-0.034) | 0.955 |  | -0.008 | (-0.059-0.043) | 0.758 |  | -0.002 | (-0.045-0.041) | 0.927 |  |
|  | Endocortical Circumference (mm) | Combined | -0.030 | (-0.033--0.027) | 0.071 | 0.864 | -0.031 | (-0.064-0.002) | 0.064 | 0.878 | -0.029 | (-0.062-0.004) | 0.081 | 0.903 |
|  |  | Males | -0.022 | (-0.079-0.035) | 0.446 |  | -0.025 | (-0.081-0.031) | 0.380 |  | -0.240 | (-0.816-0.336) | 0.414 |  |
|  |  | Females | -0.033 | (-0.081-0.015) | 0.180 |  | -0.360 | (-0.841-0.121) | 0.143 |  | -0.035 | (-0.083-0.013) | 0.156 |  |
|  | CSMI (mm4) | Combined | -0.071 | (-0.094--0.048) | <0.001 | <0.001 | -0.075 | (-0.098--0.052) | <0.001 | <0.001 | -0.069 | (-0.092--0.046) | <0.001 | <0.001 |
|  |  | Males | -0.082 | (-0.126--0.038) | <0.001 |  | -0.092 | (-0.134--0.05) | <0.001 |  | -0.089 | (-0.131--0.047) | <0.001 |  |
|  |  | Females | -0.038 | (-0.078-0.002) | 0.063 |  | -0.049 | (-0.087--0.011) | 0.012 |  | -0.045 | (-0.083--0.007) | 0.021 |  |

Associations between ACT score at 7 years and DXA and pQCT-derived bone measures in 1033 boys and 1294 girls. Data are presented for males, females and combined and show standardised regression coefficients (Beta), 95% CIs, *P* and sex interaction. Model 4: model 3 (height, age at exposure, age at outcome, maternal social class, gestational age, birth weight) + lean mass/pQCT muscle area at 17y; model 5: model 4 + fat mass at 17y; model 6: model 5 + GMS at 18 months.
